# The evolutionary and functional diversity of classical and lesser-known cytoplasmic and organellar translational GTPases across the tree of life

**DOI:** 10.1186/s12864-015-1289-7

**Published:** 2015-02-14

**Authors:** Gemma Catherine Atkinson

**Affiliations:** Institute of Technology, University of Tartu, Nooruse 1, 50411 Tartu, Estonia; Department of Molecular Biology, Umeå University, Building 6 K, 6 L University Hospital Area, SE-901 87 Umeå, Sweden; Laboratory for Molecular Infection Medicine Sweden (MIMS), Umeå University, Building 6 K and 6 L, University Hospital Area, SE-901 87 Umeå, Sweden

**Keywords:** Ribosome, GTPase, trGTPase, Translation, Molecular evolution, LUCA, EF1, EF2, IF2

## Abstract

**Background:**

The ribosome translates mRNA to protein with the aid of a number of accessory protein factors. Translational GTPases (trGTPases) are an integral part of the ‘core set’ of essential translational factors, and are some of the most conserved proteins across life. This study takes advantage of the wealth of available genomic data, along with novel functional information that has come to light for a number of trGTPases to address the full evolutionary and functional diversity of this superfamily across all domains of life.

**Results:**

Through sensitive sequence searching combined with phylogenetic analysis, 57 distinct subfamilies of trGTPases are identified: 14 bacterial, 7 archaeal and 35 eukaryotic (of which 21 are known or predicted to be organellar). The results uncover the functional evolution of trGTPases from before the last common ancestor of life on earth to the current day.

**Conclusions:**

While some trGTPases are universal, others are limited to certain taxa, suggesting lineage-specific translational control mechanisms that exist on a base of core factors. These lineage-specific features may give organisms the ability to tune their translation machinery to respond to their environment. Only a fraction of the diversity of the trGTPase superfamily has been subjected to experimental analyses; this comprehensive classification brings to light novel and overlooked translation factors that are worthy of further investigation.

**Electronic supplementary material:**

The online version of this article (doi:10.1186/s12864-015-1289-7) contains supplementary material, which is available to authorized users.

## Background

The translational GTPases (trGTPases) are an ancient superfamily of proteins, predating the last common ancestor of life (LUCA). Many trGTPases are essential for life, with core roles orchestrating the translation cycle on the ribosome (for reviews see [1-7]). The ‘classical’ trGTPases (IF2/IF5B, EF-Tu/EF1A, EF-G/EF2) are universally conserved and well studied. IF2 in bacteria, which is known as eIF5B in eukaryotes and aIF5B in archaea, is an essential initiation factor, promoting initiator tRNA binding to the small ribosomal subunit, and subsequent subunit joining. The elongation factor EF-Tu in bacteria, referred to as eEF1A and aEF1A in eukaryotes and archaea, delivers aminoacyl-tRNA (aa-tRNA) to the ribosome, while elongation factor EF-G (e/aEF2) catalyses translocation of peptidyl-tRNA across the ribosome. All trGTPases carry a highly conserved GTPase (G) domain, adjacent to a beta barrel domain [8], which together allow the phylogenetic relationships across the superfamily to be resolved. Previous sequence analysis of the P-loop superclass to which the trGTPases belong identified four families that are found in all domains of life, suggesting their presence in the last universal common ancestor of all life on earth (LUCA): EF1, EF2, IF2 and SelB, a specialized EF1-like factor for the delivery of selenocystyl-tRNA to the ribosome [8].

These four core factors have diversified during evolution by gene duplication, horizontal gene transfer (HGT) and subfunctionalisation to result in a variety of factors with different taxonomic ranges. A study of the trGTPases present in bacterial genomes identified nine subfamilies, four of which are universal (or almost universal) in bacteria: LepA, EF-G, EF-Tu and IF2 [9]. Several non-universal but broadly distributed trGTPases from bacteria, archaea, eukaryotes (both cytoplasmic and organellar) have been characterized experimentally and shown to have important roles in translation and its regulation, examples being peptide release factors RF3 and eRF3 and initiation factor a/eIF2-gamma. Some factors (such as CysN and Snu114) have diversified in function to such an extent that they may no longer interact with the ribosome [10,11].

In the absence of a comprehensive classification of the trGTPases, there are many gaps in our current knowledge about the full diversity and taxonomic distributions of the superfamily. Here, HMMs are used for sensitive sequence searching across 1483 genomes across the tree of life. From this, the evolution of trGTPases is retraced from their pre-LUCA origins though the diversification of the three domains of life and the origin of the eukaryotic organelles to the modern translational systems. The classification results identify new subfamilies and reveal the full taxonomic distribution of previously identified, although often not widely known subfamilies. Only a small fraction of the functional diversity of trGTPases has been addressed experimentally. The results of this study may be used to direct future experimental investigations, including validation of a potential Ski7 orthologue in *Candida glabrata*, rRNA RNase ability of clostridial Tet proteins, determination of function and organellar targeting of novel eukaryotic subfamilies oRF3 and mTypA, testing of ribosome binding capabilities of Snu114 and CysN, and Dom34-binding and ribosome rescue abilities of GTPBP1 and aGTPBP. The results identify factors that are only limited to certain lineages, suggesting lineage-specific translational control mechanisms that exist on a base of core factors.

## Results and discussion

### trGTPases through the diversification of life on earth

The identification of all distinct subfamilies of trGTPases was an iterative process beginning with BlastP searching of known trGTPases against a set of genomes selected broadly across the tree of life. This was followed by subsequent rounds of phylogenetic analysis to identify clusters representing subfamilies, and sequence searching with Hidden Markov Model (HMM) profiles of trGTPase subfamily alignments against a set of 1483 genomes. This led to the final identification of 57 distinct trGTPase subfamilies (Table 1). The taxonomic distribution of the identified subfamilies supports the presence of at least EF1, EF2, SelB and IF2 in LUCA. These progenitor trGTPases subsequently diversified into 14 bacterial, 7 archaeal and 35 eukaryotic (of which 21 are known or predicted to be organellar) subfamilies. The complement of trGTPases that can be found in each of the genomes considered here is recorded in the additional files available online: all identified trGTPases and their sequence identifiers (Additional file 1) and lists of trGTPases found in each genome, sorted by taxonomy (Additional file 2). As there are inconsistencies in the naming of trGTPases, a table of synonyms is included in Additional file 3.

**Table 1:** **trGTPase orthologue presence across the cytoplasm of bacteria, eukaryotes and archaea, and eukaryotic organelles**

	**Cytoplasmic**			**Organellar**		
	**Bacteria**	**Archaea**	**Eukaryotes**	**Mitochondrion**	**Plastid**	**Unknown**
**EF2**	EF-G	aEF2	eEF2		cEFG/apiEFG	
	spdEFG1			mEFG1		
	spdEFG2/lEFG2			mEFG2		
	gcEFG2					
	EFGII					
	Tet					
	RF3					oRF3
	TypA			mTypA/exTypA	cTypA	
	LepA			mLepA	cLepA/apiLepA	
			Ria1			
			Snu114			
**EF1S**	EF-Tu	aEF1A	eEF1A	mEFTu1	cEF-Tu/apiEFTu	
				mEFTu2		
	actEFTu2					
			EFL			
			Hbs1			
			Ski7			
			eRF3			
			eRF3-2			
	CysN					
		aGTPBP	eGTPBP/eGTPBP1			
			eGTPBP2			
		aIF2g	eIF2g			
	SelB	aSelB	eSelB			
		aSelBL				
**IF2**	IF2	aIF5B	eIF5B	mIF2	cIF2/apiIF2	
				mIF2-2		
				hIF2		

**Figure 1:**
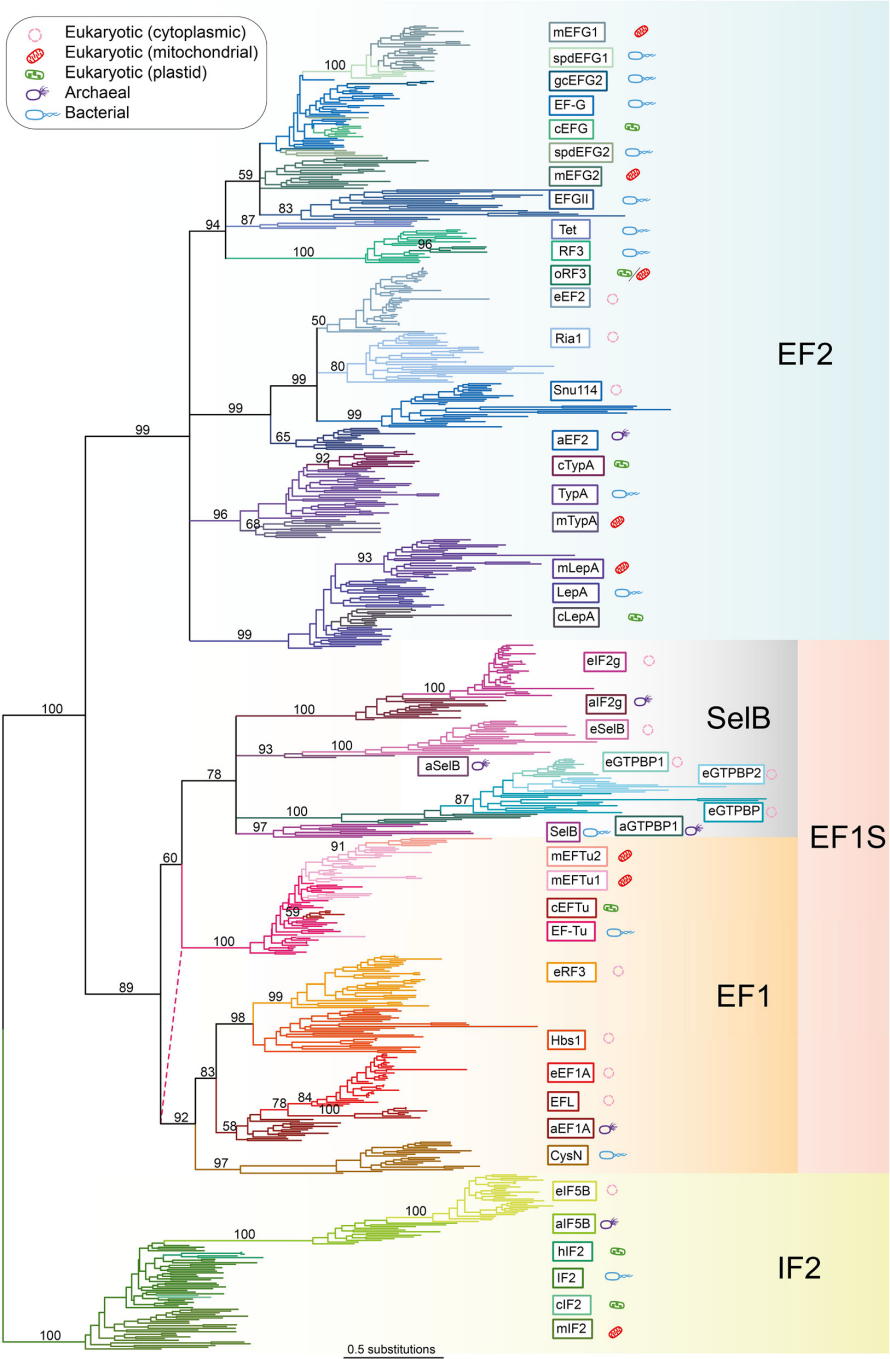
**The trGTPase superfamily tree.** The tree shown is an unrooted maximum likelihood phylogeny of trGTPase subfamilies from across the tree of life. Numbers on branches show bootstrap support from 100 replicates. Nodes separating subfamilies with less than 50% bootstrap support have been collapsed. The pink dotted line shows an alternative position for the clade containing bacterial and organellar EF-Tu, as supported by operon structure. Branch lengths are proportional to the number of amino acid substitutions (see lower scale bar). The icon next to the subfamily name indicates the domain of life and known or predicted subcellular compartment in which that trGTPase is found, as per the inset box.

Phylogenetic analysis of sequences that can be unambiguously aligned across the whole superfamily gives a tree with a tripartite structure, with clear divisions corresponding to the EF1S family (so called here because it comprises EF1 and SelB midfamilies), the EF2 family and the IF2 family (Figure 1). The term midfamily is used here to describe strongly supported clusters of subfamilies within a family that have representatives in all domains of life. The tree shows weak support for the grouping of the EF-Tu subfamily with the SelB midfamily (maximum likelihood bootstrap support of 60%, Figure 1). However, in addition to their conserved functional roles, EF-Tu and aEF1A can both be found in the same *str* operon structure [12], suggesting orthology. Therefore, the association of EF-Tu with the SelB mid-family is likely to be an artifactual relationship, with EF-Tu more likely being a component of the EF1 family. The unexpected association of EF-Tu with the SelB midfamily may be a result of homoplasy; for instance bacterial EF-Tu sequences and bacterial SelB sequences may be evolving convergently due to similar functional constraints resulting from their similar roles in tRNA delivery to the bacterial ribosome. Indeed, phylogenetic analysis of the EF1 family with bacterial SelB and other long branches subgroups excluded shows a relationship of EF-Tu with a/eEF1A to the exclusion of aIF2g and aSelB, albeit with low statistical support (maximum likelihood bootstrap support of 51%, Additional file 4).

**Figure 2:**
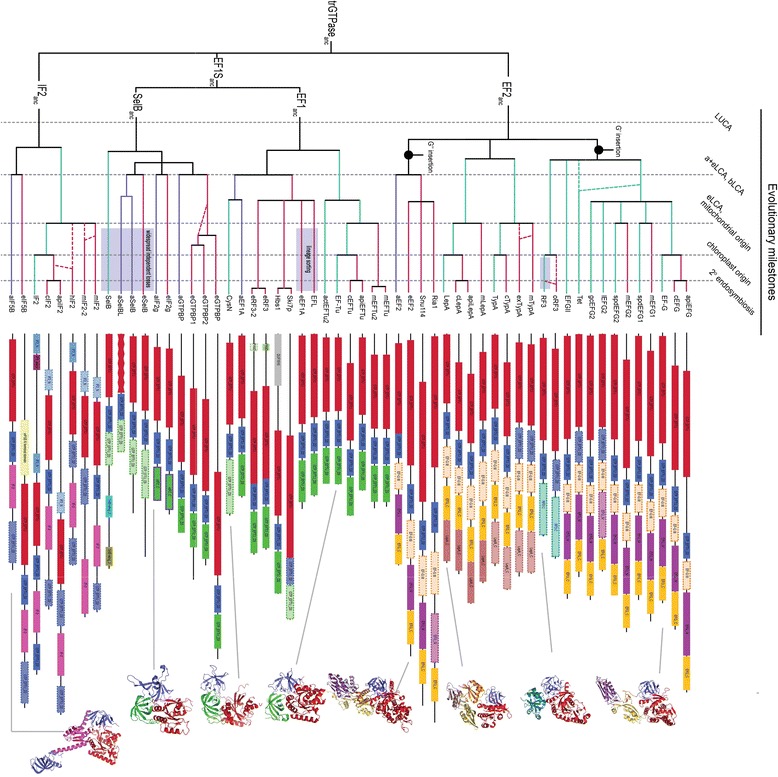
**Relative timeline of trGTPase diversification.** The diagram summarizes evidence from phylogenetic relationships, domain architecture, transit peptide prediction and taxonomic distributions to show the relative divergence times of trGTPase families and subfamilies. Vertical dotted lines indicate major milestones in the evolution and diversification of life on earth, while horizontal branches are lineages of trGTPases in bacteria (green), archaea (blue) and eukaryotes (red). The subscript protein name suffix “anc” stands for ancestral. The tree assumes that archaea and eukaryotes share a common relative to the exclusion of bacteria. Branch lengths and time between ancestors are not to scale. Branches with dashed lines show uncertainties in relationships, and shading shows cases of particularly high lineage specific loss. LCA stands for last common ancestor, with bLCA being the ancestor of bacteria, eLCA being the ancestor of eukaryotes, aLCA being the ancestor of archaea, and a+eLCA being the ancestor of all archaea and eukaryotes. Typical subfamily domain structures are shown to the right of the tree. Boxes with solid borders show domains that are predicted with PFam. Where the domains are present but do not hit PFam HMMs, the boxes are shown with dotted borders. The G domain (Pfam name GTP_EFTU) of aSelbL is shown with an undulating border to indicate particular divergence in this subfamily. Protein structures are shown on the far right, and are linked with a grey line to their respective subfamily. Protein Data Bank IDs for the structures are as follows: EF-G: 1DAR [14], RF3: 2H5E [15], LepA: 2YWE, eEF2: 1N0V [16], EF-Tu: 1EXM [17], CysN: 1ZUN [18], aIF2g: 3PEN, aIF5B: 1G7R [19].

The pattern of presence and absence of all the trGTPase subfamilies in the genomes considered here allows the diversifications of trGTPase lineages and protein architecture to be mapped on to a relative timeline of five major milestones in the evolution of life on earth: 1) the lifetime of LUCA, 2) the origin of bacteria (the bacterial last common ancestor, bLCA) and the ancestor of eukaryotes and archaea (a+eLCA); these are summarized into one milestone given the uncertainty in the relative timing of these events, 3) the origin of eukaryotes and the endosymbiotic event that gave rise to the mitochondrion (also grouped into one milestone as these two events may be connected [13]), 4) the origin of the chloroplast and 5) the secondary endosymbiosis event that gave rise to the apicoplast of alveolates (Figure 2). In addition to the core G domain and domain II, each family has innovated its own particular domains; the EF1 family evolved its domain III (Pfam name GTP_EFTU_D3), the EF2 family evolved domains III, IV (EFG_IV) and C (EFG_C), and the IF2 C terminal domain (Pfam name IF-2) evolved in the IF2 family. There have also been within-family domain developments: the LepA C terminal domain (LepA_C) evolved in bacteria in the lineage to LepA and TypA, SelB evolved C terminal extensions specific to eukaryotes and bacteria, eukaryotes redeveloped the N terminal regions of Hbs1p, Ski7p and the eRF3 paralogues, bacteria evolved a particular N terminal region of IF2 (IF2_N), and a whole new domain evolved in the C terminus of bacterial RF3 (Figure 2).

### The functional diversity of trGTPases: a tour by family

#### EF1

The core, and most likely ancestral function of EF1 is delivery of aa-tRNAs to the ribosome [20]. This function is carried out by EF-Tu, eEF1A (or EFL) and aEF1A in bacteria, eukaryotes and archaea, respectively. EF-Tu is one of the most abundant bacterial proteins [21] and is unusual in that it is present as two copies in most bacteria (coded by the *tufA* and *tufB* genes), which are often identical, or nearly identical in sequence. This is a result of gene conversion by homologous recombination between both genes [22-24]. Only one EF-Tu-like subfamily (actEF-Tu found in some actinomycetales, Additional files 1 and 2) is clearly distinct from EF-Tu and suggests that gene conversion is non-functional between *tufA* and *tufB* in these organisms. In *Streptomyces*, these duplicates appear to be condition-specific translation factors, being expressed during stress conditions [25] and showing resistance to antibiotics targeting EF-Tu [26]. Most actinomycetales only carry one EF-Tu however, suggesting that in the absence of gene conversion, duplicate copies of EF-Tu can readily be lost.

Mitochondriate eukaryotes encode EF-Tu in their nucleus, which is subsequently transported to the mitochondrion. Mitochondrial EF-Tu has been reported to have undergone duplication in nematodes, with the resulting paralogues evolving to become specialized for different structures of tRNAs, lacking the D or the T arm [27-29]. Searching with HMMs of both these versions of EF-Tu show that these duplicates are also present in Annelida, Mollusca and Arthropoda, suggesting this duplication occurred in the lineage to protostomia (Figures 1 and 2, Additional files 1 and 2).

There are three ancient paralogues of eEF1A in eukaryotes: eRF3, Hbs1 and EFL (Figures 1 and 2). eRF3 and Hbs1 are well characterized experimentally. eRF3 associates with the class I release factor eRF1, which recognizes the stop codon and onsets termination of termination [30]. eRF3 and eRF1 also have an additional role in mRNA quality control, triggering nonsense-mediated mRNA decay in response to a premature stop codon [31-33]. Hbs1, which binds eRF1 paralogue Dom34 is also an mRNA decay factor, in this case triggering “no-go” mRNA decay upon ribosomal stalling [21]. In yeast, Ski7 – a relatively recent paralogue of Hbs1 – is required for “non-stop” decay where the ribosome fails to terminate and reads through to the poly-A tail of the mRNA [33]. This factor was thought to be limited to the Saccharomyces genus [34]. Surprisingly however, this study has identified a divergent paralogue of Hbs1 in *Candida glabrata* (accession number XP_448465.1), which is classified as Ski7 by the HMM models. Experimental validation is required to establish whether this is a functional equivalent of Ski7. Recently, NSD functions have been assigned to Hbs1:Dom34 in mammals [35]. This suggests an ancestral role of Hbs1 in both types of rescue, with this function being partitioned between Hbs1 and Ski7 upon duplication in yeast. Indeed, Hbs1p from *Saccharomyces kluyveri*, a yeast that does not carry Ski7p, can complement an S*. cerevisiae* Ski7p deletion mutant [36].

eRF3-2 is an animal-specific duplication of eRF3, found in Chordata, Cnidaria and Echinodermata (Additional file 2). The two paralogues are highly similar in sequence, with differences mainly being found in the variable N terminal domain [37,38]. eRF3 rather than eRF3-2 appears to be the primary translation termination factor as only silencing of the former induces a significant increase in stop codon readthrough [39]. The N terminal regions of all the eukaryotic paralogues of EF1 have N terminal extensions that vary in length and sequence composition both among and within subfamilies. These appear to be important for lineage-specific inter-molecular interactions, such as the binding of poly-A binding protein PABP via a PAM motif in metazoan eRF3a/b (Figure 2, [37]).

EFL is one of the most enigmatic trGTPases. Despite being a divergent paralogue of eEF1A, its function appears to be identical to the classical elongation factor as its presence is almost entirely mutually exclusive with eEF1A (Additional file 2 [40,41]). EFL presence is also strongly associated with absence of eEF1Bα, the factor responsible for recharging eEF1A with GTP, suggesting that similarly to other EF1S family member SelB, EFL is able to self-recharge [40]. The unusual broad but non-continuous distribution of EFL has been explained by both HGT and long term co-maintenance followed by lineage specific loss (For example [41-43]). The timing of EFL’s origin by gene duplication is unknown, but may have been early in eukaryotic evolution, given its deep placement as a sister group to eEF1A (84% BP, Figure 1).

CysN is the only clear case of HGT giving rise to an entirely new subfamily of trGTPases. This subfamily results from an ancient transfer of aEF1A from archaea to bacteria [44]. CysN is a component of the ATP sulfurylase (ATPS) complex, which acts in the first step of the sulfate metabolism pathway, a process crucial for the biosynthesis of sulfur-containing amino acids and cofactors. GTP hydrolysis by CysN drives the activity of the CysD subunit, producing adenosine-5′-phosphosulfate (APS) from ATP and sulfate. In the next step of the pathway, APS is phosphorylated by the protein CysC. In various bacteria, CysN is found fused to CysC, the adjacent gene in the CysDNG operon [18].

#### SelB

Although not universal, the widespread distribution of SelB in bacteria, archaea and eukaryotes suggests it was present in LUCA along with EF1, EF2 and IF2 (Figure 2). Like EF-Tu/EF1, SelB functions in aminoacyl-tRNA delivery, but is specific for the incorporation of the amino acid selenocysteine at recoded stop codons [45]. The signal for selenocysteine incorporation, the mRNA SECIS element, is recognized by bacterial SelB via its mRNA-binding C terminal domain. In eukaryotes this interaction is mediated by a separate protein, SBP (SECIS-binding protein). It is currently unclear how archaea recognize the SECIS element, as an orthologue of SBP is absent in archaea, and the aSelB CTD is unconserved with that of bacteria and eukaryotes [46,47] (Figure 2). Widespread independent losses in multiple lineages are observed with SelB, as with other components of the selenocysteine incorporation machinery [48]. In archaea, a duplication of aSelB has given rise to aSelBL, a factor with unknown function, a distribution broader than that of aSelB, and a G domain that is disrupted to varying degrees [49].

A duplication of SelB appears to have given rise to e/aIF2-gamma (referred to here as e/aIF2g) in eukaryotes and archaea (Figures 1 and 2). This factor is a subunit of the eIF2 initiation factor in eukaryotes, and is responsible for recognizing and delivering the initiator tRNA to the ribosome, while also scanning for the start codon and its context, hydrolysing GTP upon start codon recognition [50]. In archaea, aIF2g has a second function in counteracting 5′ mRNA decay [51], a function that may not be required of eIF2g, due to the stabilizing effect of the 5′ CAP on eukaryotic mRNAs.

The GTPBP trGTPases form a relatively divergent group (Figure 1) and are particularly variable in their N terminal regions. aGTPBP is widespread in archaea, being found in Euryarchaeota, Korarchaeota and Crenachaeota, although not identified in Nanoarchaeota or Thaumarchaeota. A GTPBP-like protein is represented in all major groups of eukaryotes, although it is not universal within groups (Additional file 2). Many eukaryotes have two copies of these proteins: eGTPBP1 and eGTPBP2, where these can be distinguished. This suggests a duplication event predating the eukaryotic last common ancestor (LCA). There is not enough phylogenetic resolution to confidently classify all protist duplicates as either eGTPBP1 or 2, therefore some are simply classified as eGTPBP (Additional files 1 and 2). Despite their widespread distribution, the function of the GTPBPs has been largely obscure, with information mainly limited to expression and knock out analyses in mice [52,53]. More recently, however, eGTPBP1 was found to associate with the exosome to enhance mRNA decay [54] and eGTPBP2 bound to Dom34 was found to relieve ribosome stalling caused by non-functional tRNAs [55]. Thus, the GTPBPs may represent a family of ribosome rescue proteins with roles in mRNA surveillance similar to those of other EF1 family members Hbs1 and Ski7. Interestingly, there are no GTPBP representatives in yeasts, although other Ascomycete fungi carry GTPBP1 and GTPBP2 (Additional file 2). Phylogenetic analysis of GTPBPs shows particularly long branches for the Ascomycete GTPBP2 factors, suggesting possible divergence towards loss in this lineage (Additional file 5). Multicellular plants do not carry any GTPBP, although green algae have two GTPBPs perhaps originating from eGTPBP1 and eGTPBP2 (Additional files 2 and 5).

#### EF2

EF-G (e/aEF2) is the universal, essential factor that catalyses the translocation of peptidyl-tRNA from the A to the P site of the ribosome and forms the core of the EF2 family. In bacteria, EF-G has a second function promoting ribosome recycling via subunit splitting in concert with the ribosome recycling factor RRF [56-58]. Within bacteria, there are multiple paralogues of the EF2 family, the most distinctive being TypA, LepA, Tet, RF3, EFGII, gcEFG, spdEFG1 and spdEFG2. With the exception of TypA and LepA, the bacterial and organellar members of the EF2 family carry an insertion in the G domain relative to EF1/IF2. This is referred to as the G’ domain. The eukaryotic and archaeal a/eEF2 factors share a non-homologous insertion in a different location of the same domain, referred to as the G” subdomain (Additional file 6).

Both LepA and TypA are widespread in bacteria, but non-essential, suggesting they are condition-specific factors. They share a common (although divergent) C terminal domain in addition to lacking both the G’ or the G” subdomains (Figure 2, Additional file 6). Along with the phylogeny, which places them outside of both the bacterial and archaeal-eukaryotic lineages (94% and 99% MLBP respectively, Figure 1), the atypical domain structures of LepA and TypA suggest they diverged at a very early point in EF2 family evolution. In fact, the possibility that they were present in LUCA and then lost in the archaeal-eukaryotic lineage cannot be ruled out. The functions of these proteins have not been entirely resolved. LepA, also known as EF4 has been shown *in vitro* to promote back translocation, that is reverse movement of peptidyl-tRNA and deacylated tRNA from the P and E sites to the A and P sites, opposite to the movement catalysed by EF-G [59]. It has also been argued that LepA’s main function may be in sequestering ribosomes in an intermediate conformational state of translocation, leading to transient elongation pausing [60]. Such pauses in the rate of elongation may aid co-translational folding of the nascent peptide chain [61]. Structures of LepA on the ribosome are also consistent with back translocase or ribosome sequester function [62,63]. While the physiological significance of LepA remains uncertain due to a lack of a distinct phenotype upon deletion [64], it appears to be part of a response to extreme conditions such as temperature and ionic stress [61,65]. There are also mitochondrial and chloroplast versions of LepA; mLepA is almost universal in eukaryotes carrying this organelle (the main exception being apicomplexan parasites) while cLepA appears to be universal in archaeplastida (Additional file 2). Both mLepA and cLepA are important for organellar translation under suboptimal conditions [66,67].

Less is known about TypA (also known as BipA), except that it is ribosome associated [68,69], and like LepA seems to be involved in environment and stress response in bacteria [65,70] and chloroplasts [71]. It is also important for virulence of bacterial pathogens [72]. The results of the present study identify a previously unreported TypA subfamily (mTypA) in some Archaeplastida, Amoebozoa and fungi (Additional files 1 and 2). Where transit peptides are predicted for this subfamily, they are mostly mitochondrial (Additional file 7). A distinct group in excavates – exTypA –does not cluster with mTypA, but is also mitochondrially targeted according to transit peptide predictions (Additional files 1, 2 and 7).

Tet is the subfamily representing the TetM/TetO-like group of tetracycline resistance proteins. Tet proteins have the same domain structure as EF-G, but the three loops that form the tip of domain IV and interact with peptidyl-tRNA in EF-G [73] are differentially conserved in Tet proteins. It is these loops of Tet that interact with and dislodge tetracycline from the ribosome [74,75]. The discontinuous taxonomic distribution (Additional file 2) suggests multiple HGT events within the Tet subfamily, an observation unsurprising for an important antibiotic resistance gene. In fact, some of the Tet proteins are mosaic sequences as a result of homologous recombination among paralogues [76]. Tet proteins from ten species of clostridiales have acquired an additional domain, the YacP-like NYN RNAse domain (Additional file 8). This domain has been proposed to be involved in maturation of rRNA and tRNA [11] and may represent a novel mechanism of action of antibiotic resistance by these Tet proteins that involves RNase activity.

EFGII is widespread in bacteria with a divergent G domain and unknown role [77,78] (Additional files 2 and 6). However, it still retains some of EF-G’s original function as it can substitute for a deletion of EF-G, carrying out translocation at a much slower rate [77]. The release factor RF3 has a non-continuous but broad distribution in nearly all known bacterial phyla, suggesting it may have been present in the bLCA (Figure 2, Additional file 2). However, a great deal of lineage-specific loss is observed for this protein that was originally called a classical factor [9]. RF3 is best known for its involvement in translation termination, promoting the release of the stop codon recognizing RF1 and RF2 release factors from the ribosome [79,80]. An additional role of RF3 was recently discovered in post-peptidyl-transfer quality control, where peptides carrying mistakes are prematurely terminated [81,82]. This appears to operate via RF3’s association with RF2, rather than RF1. Among the EF2 family members, RF3 is unusual in that it does not carry domain V (Pfam name EFG_C), and instead has its own unique C terminal domain with a novel fold [15] (Figure 2).

While the above bacterial factors in the EF2 family appear to be ancient paralogues of EF-G, the factors gcEFG2, spdEFG1 and spdEFG2 are duplications *within* the EF-G family. gcEFG2 is a second copy of EF-G with unknown function found in eight species of cyanobacteria, nine species of Alphaproteobacteria, two species of Betaproteobacteria, 45 Gammaproteobacteria and one Verrucomicrobium. spdEFG1 and spdEFG2 have a largely mirrored taxonomic distribution in spirochetes, planctomycetes and Deltaproteobacteria [83]. The current analysis has also identified spdEFG1 in *Fibrobacter* (Fibrobacteres) and *Cyanothece* and *Nostoc* (Cyanobacteria), and spdEFG2 in *Stenotrophomonas* and *Allochromatium* (Gammaproteobacteria), *Bradyrhizobium* (Alphaproteobacteria) and some Actinobacteria that also carry actEF-Tu2 (Additional files 1 and 2). It is tempting to speculate that starvation-induced actEFTu2 and spdEFG2 work together in Actinomycetes as stress-specific elongation factors. The spdEFG1 and spdEFG2 groups have also given rise to mtEFG1 and 2 in mitochondria, and are subfunctionalised for the two EF-G functions in translocation and ribosome recycling [83-85]. lEFG is a divergent group of EF-Gs found in the *Leptospira* genus of spirochetes, and is probably the hitherto unidentified spdEFG2 orthologue in these organisms. It should also be noted that relatively recent duplications and HGT events are observable within the EF-G subfamily in fine-scale analyses [79], but HMMs are not able to separate them into distinct groups and therefore they are classified as additional EF-Gs in Additional files 1 and 2.

There have been two duplications of eEF2 that predate the last common ancestor of eukaryotes, giving rise to Ria1 and Snu114 (Figure 2). Ria1, also known as EFL1 (not to be confused with EFL) appears to be universal in eukaryotes (Additional file 2), and is a ribosome biogenesis factor [86]. It probes the integrity of the P site of the 60S subunit and promotes the release of eIF6 upon recognition of a correct fold [87,88]. Snu114 is a spliceosome factor involved in U4/U6 unwinding during spliceosome assembly [10,89-91]. It also has a broad distribution across eukaryotes, only lacking in Microsporidia, *Bigelowiella* and *Giardia* in this study.

#### IF2

IF2 is the only family with a single cytoplasmic orthologue; Only organellar IF2 appears free to duplicate (Figures 1 and 2). This factor – IF2, eIF5B and aIF5B in bacteria, eukaryotes and archaea respectively – is required for promoting initiator tRNA binding to the small ribosomal subunit, and the recruitment of the large subunit in order for translation to begin [92,93]. Relative to aIF5B, the N terminal regions of eIF5B and IF2 are extended, varying in sequence and length (Figure 2). Structural analyses of these orthologues have suggested wildly different conformations, indicating significant flexibility and raising the possibility that the universal functions of IF2/a/eIF5B may be mediated by different mechanisms in different domains of life [19,94-97]. Surprisingly, a duplicate domain II-homologous region is found in the C terminus of IF2/a/eIF5B (Figure 2). This second domain II (referred to as domain IV) interacts with the CCA-end of Met-tRNA on the ribosome. It is probably no coincidence that the homologous domain II of EF-Tu interacts with the CCA-end of aminoacyl tRNA [98].

The mitochondrial and chloroplast-targeted IF2s (mIF2 and cIF2, respectively) are universal in organisms that encode those organelles. A sequence insertion in mIF2 was previously proposed to compensate for the function of IF1, absent in all mitochondria. However, more thorough sequence analysis revealed that the full conserved form of the insertion is limited to vertebrates, and is not in fact a universal attribute of mIF2 [99]. mIF2-2 is a duplication of mIF2 found in some excavates and apicomplexa [99]. The haptophyte specific hIF2 may be another duplication of mIF2, or may be a chloroplast IF2 (cIF2) duplication (Figure 2).

### Organellar trGTPases

Many nuclear-encoded organellar trGTPases have been identified in the proteomes of their respective subcompartment. Some of these have been well characterized to various extents since their discovery (cEF-G, mEF-G1 and 2, cIF2, mIF2 cTypA, mLepA and cLepA). As well as revealing the taxonomic distribution of these factors (Additional file 2), the current study has identified two additional organellar trGTPases not previously reported: organellar RF3 (oRF3) and mitochondrial TypA (mTypA and exTypA). The oRF3 factor is only found in plants, suggesting it may be a chloroplast factor. This is also supported by the oRF3 HMM model hitting cyanobacteria sequences with greater significance than the general bacterial RF3 model. However, a chloroplast transit peptide is only predicted for *Fragilariopsis cylindrus* oRF3, and mitochondrial targeting peptides are predicted for *Aureococcus anophagefferens*, *Ectocarpus siliculosus*, *Selaginella moellendorffii* and *Volvox carteri* (Additional file 2). Additionally, oRF3 is not identifiable in the published proteomes of the *Chlamydomonas reinhardtii* mitochondrion [100] or chloroplast [101]. Thus, the subcellular target of oRF3 remains to be determined, and may be differently localized in different taxa.

Mitochondrial targeting is predicted for most mTypA and exTypA proteins (Additional file 7). The exceptions are *Toxoplasma gondii*, *Phaeodactylum tricornutum* and *Ostreococcus sp*. mTypA for which potential chloroplast targeting is also predicted. There are mTypA representatives across the eukaryotic tree of life: opisthokonts, alveolates, haptophytes, plantae and possibly excavates in the form of exTypA. Therefore, although it has been lost independently in multiple lineages, mTypA was probably in the bacterial ancestor of the mitochondrion. This suggests that the mitochondrial ancestor had a rather full complement of trGTPases: at least LepA, TypA, spdEFG1, spdEFG2, EF-Tu and IF2.

The chloroplast factors are often so similar in sequence to the orthologous bacterial factors that they can not be distinguished with HMMs, and sometimes even with phylogenetic analysis; cyanobacterial sequences sometimes fall within mainly plant-containing clades and have more significant E values for the plant models than general bacterial model (Additional file 1). The translation system of chloroplasts is in general more similar to the bacterial system than is the mitochondrial system, which can easily be distinguished from bacterial sequences with HMMs. This may reflect the more recent acquisition of the chloroplast in eukaryotes (in the lineage to green plants [102]) than the origin of the mitochondrion (perhaps predating the last common ancestor of extant eukaryotes [103].

Chloroplast trGTPases also have apicoplast equivalents (plastid factors with an ‘api’ prefix in Table 1). The apicoplast is the photosynthetic organelle of apicomplexan parasites such as *Plasmodium* and *Toxoplasma*, which was acquired via secondary endosymbiosis [102]. Apicoplast-functioning proteins are highly divergent and therefore have been excluded from the universal trGTPase phylogeny of Figure 1. While most organelle-targeted trGTPases are encoded in the nucleus and post-translationally targeted to their respective organelle, EF-Tu is an exception. The *tufA* gene encoding EF-Tu is located in the apicoplast genome in some apicomplexa [104], and the mitochondrial genome in some Jakobid excavates [105].

### Conservation of G domain active site residues

The alignment of the G domain and domain II, which are common to all trGTPases shows that the strongest conservation is found in the nucleotide-binding loops of the GTPase active site (Additional file 1). It was recently suggested that with the exception of eIF2g, all trGTPases use monovalent cations (M^+^) as structural co-factors that stabilize the GTP-bound (active) state [106]. M^+^ binding is mediated by Asp in the P loop and Gly in Switch I. These amino acids are substituted for Ala and Asn in eIF2g, explaining its lack of M^+^ binding (Additional file 1) [106]. The results here show additional variation in the M^+^ binding sites; EFGII carries Gly in the P loop, and EFGII, Ria1, Snu114, eEF2 and aIF5B show a lack of conservation in the M^+^ binding residue of Switch I. This raises the question of if, and how these trGTPases use M^+^ as a stabilizing cofactor.

### Divergence and convergence in trGTPase evolution

Only two trGTPases (CysN and Snu114) have reported functions that do not involve ribosome binding or translation. However, additional ribosome-associated roles cannot be ruled out for these proteins, given their conservation of domain architectures with other trGTPases (Figure 2). The close relative of Snu114, Ria1 appears to exert its ribosome binding function not in translation per-se, but in the preceding step, ribosome biogenesis. This role is also associated with other GTPases that are more distant relatives of the trGTPases, such as ERA [107], Der [108] and ObgE [109].

In contrast to this functional divergence, convergence of function is also seen in the superfamily, specifically concerning binding of tRNA, and structural mimics of tRNA. IF2 and e/aIF2g have converged in molecular binding function, having evolved initiator tRNA recognition in parallel. Similarly RF3 and eRF3 have independently evolved to interact with tRNA-mimicking class I release factors RF1/2 and eRF1 that identify stop codons and promote peptide release. Eukaryotic EF1 family members Hbs1 and eGTPBP have seemingly independently evolved roles in ribosome rescue via interactions with the eRF1 paralogue Dom34 [21,55]. However, Dom34 interaction appears to be an ancestral function of the EF1 family, as it is aEF1A that associates with Dom34 to carry out no-go decay in archaea [110]. Thus, the ability to interact with Dom34 may be a feature retained throughout EF1 family evolution, a hypothesis that is readily testable.

### Duplicability of trGTPases

The trGTPases differ in their duplicability among families and domains of life. With the exception of the organellar homologs and species-specific isoforms, the IF2 family shows very low duplicability, being comprised of a single orthologue (IF2/a/eIF5B, Figures 1 and 2). The reason for this is unclear. The high connectivity of information processing proteins such as trGTPases in core interaction networks is a potential barrier to gene duplication [111]; duplicates alter the stoichiometry of binding partners and can lead to mis-interactions. Preventing such mis-interactions may be particularly important in initiation, which is arguably the most tightly regulated step of translation [112]. Overlapping, leaky functions may not be tolerated for IF2 as they are with EF2 family proteins; for example it is unlikely that EF-GII’s main function is as a translocase but nevertheless, it can translocate in the absence of EF-G [77].

Another important factor in whether a duplicate will be retained is whether the original protein is multifunctional; proteins with more than one function can be subfunctionalised upon duplication [113]. eEF1A has numerous moonlighting functions and has given rise to a variety of paralogues [114]. eRF3 and Hbs1p are paralogues of eEF1A with specialised functions that in archaea are still carried out by aEF1A. Such a variety of functions have not been reported for IF2, and this specialization may have hindered the diversification of the family.

EF-G is the most duplicated trGTPase in bacteria, with at least eight duplications being apparent. This is in contrast to two, one (or two if you include CysN) and zero bacterial duplications in EF1, SelB and IF2 families, respectively. The pattern is different in eukaryotic cytoplasmic translation, where EF1 is the biggest source of functional innovation (two, five, three and zero duplications in EF2, EF1, SelB and IF2 respectively).

Many multicellular organisms have an abundance of single-species-specific trGTPase paralogues for cytoplasmic subfamilies (Additional files 1 and 2). In animals, these are multiple protein isoforms of almost identical sequences, sometime with varying lengths. An example of this are the two isoforms of human eEF1A that are differentially post translationally modified and expressed [115]. In plants, such duplicates may be due to polyploidy events. Some protists have also experienced species-specific massive duplication of their core trGTPases. Examples include duplicates of cytoplasmic and organellar sequences in ciliates *Perkinsus marinus* and *Paramecium tetaurelia*, and the several versions of eEF1A and eEF2 in excavate *Naegleria gruberi*. Unlike the mammalian isoforms, these protist versions are often highly divergent in sequence.

Archaea have not duplicated their trGTPases to the extent of eukaryotes and bacteria. Assuming archaea and eukaryotes share a common ancestor (but without making any assumptions about whether these two domains are sister groups, or whether archaea are paraphyletic to eukaryotes), there were six trGTPase factors in the a + eLCA (EF2, EF1, IF2g, SelB, GTPBP and IF5B). From those six trGTPases present in the common ancestor of eukaryotes and archaea, archaea have only added one more protein to their repertoire: aSelBL. Meanwhile, eukaryotes have added six more cytoplasmic factors: eGTPBP2, eRF3, Hbs1, EFL, Ria1 and Snu114. The lack of duplicability in archaea may be due to a general tendency for genome streamlining in this domain of life [116].

The minimal trGTPase composition is three, four, and six in bacteria, archaea and eukaryotes, respectively (Additional file 2). The three bacterial factors EF-Tu, EF-G and IF2 are found alone in the obligate endosymbionts *Candidatus Carsonella ruddii*, *Candidatus Hodgkinia cicadicola* and *Candidatus Sulcia muelleri*. Suprisingly, the free-living bacterium *Mycoplasma crocodyli* also manages to survive with just those three factors. In archaea, *Aciduliprofundum boonei* and *Aeropyrum pernix* carry just aEF1A, aEF2, aIF2g and aIF5B, while in eukaryotes, the microsporidia *Encephalitozoon cuniculi* and *Encephalitozoon intestinalis* have the most streamlined composition with eEF1A, eEF2, eIF2g, eIF5B, eRF3 and Ria1.

In the EF2 and EF1S families, duplications have resulted in paralogues that are variations upon common functional themes. The EF2 family “theme” is the promotion of conformational changes by the ribosome in order to trigger the displacement of another molecule. For example EF-G/e/aEF2 catalyse translocation of peptidyl-tRNA, Tet proteins promote displacement of tetracycline, and RF3, the release of class I RFs. Duplications in the EF1S family on the other hand have mostly resulted in factors specialized for binding certain tRNAs or structural mimics of tRNAs; EF-Tu/e/aEF1A deliver animoacyl-tRNAs to the ribosome while GTPBP2, eRF3, Hbs1 (and possibly Ski7) and aEF1A bind tRNA mimics eRF1 and Dom34 [21,30,55].

Taken together, the combined evidence suggests that the last common ancestor of all life on earth (LUCA) had at least four trGTPases: IF2 for promoting initiator-tRNA binding and subunit association, EF1 for aminoacyl-tRNA delivery to the ribosome, EF2 for translocation of peptidyl-tRNA from the ribosomal A site to the P site, and SelB for the specialized delivery of selenocystyl-tRNA to the ribosome and decoding of the SECIS insertion element (Figure 2). However this may well be an underestimate of LUCA’s complement of trGTPases; LUCA is not necessarily a primitive organism, but is rather the last organism that we can trace back to from current sequence data from extant organisms. Major lineages of trGTPases may have been lost as well as gained pre- and post-LUCA. Indeed, the analysis here shows multiple cases of widespread independent losses of trGTPases. Perhaps the most striking examples of loss are in the bacterial RF3 subfamily and across the whole SelB family (Figure 2).

## Conclusions

The trGTPases are at the core of translation and its regulation in all domains of life, and have been since before LUCA. On a base of essential trGTPases, additional factors have evolved by duplication and divergence to control translation on an organism-, organelle- and environment-specific basis. The genomic profile of classic, lesser-known and entirely novel trGTPases presented in this study opens avenues for experimental investigations and is a step towards understanding mechanisms of protein synthesis on a system-specific level.

## Methods

### Initial sequence searching

To uncover the general diversity of trGTPases and generate sequence alignments for initial HMMs, 38 known trGTPases from the four major families (EF1, EF2, SelB and IF2) were used as queries in local BlastP v2.2.25+ [117] searches with an E value limit of 1e-3, against a set of 66 genomes across the tree of life: 27 eukaryotes, 26 bacteria and 13 archaea (Additional files 9 and 10). The 38 query trGTPases were identified through literature searches, and sequences were retrieved mainly from Uniprot [118] and some from RefSeq [119] (Additional file 9). Bacterial and archaeal genomes were selected primarily to sample across the full diversity (one genome per phylum and one per class in the case of bacteria and archaea respectively), and secondarily by genome size, as some trGTPases may be absent in particularly reduced genomes. The eukaryotic sequences were selected to sample broadly across the eukaryotic tree of life (Additional file 10).

In order to avoid false positive non-trGTPase hits generated by similarity to other domains, all the hit sequences were scanned online against the Pfam database [120] and only those sequences retaining the trGTPase G domain model GTP_EFTU were retained. The G domain regions of the hits were extracted and aligned using MAFFT v6.964b [121]. To reveal the structure of the trGTPase family tree, an initial tree was made with RAxML version 7.3.0 [122] on the Cipres portal [123] using the LG model and 100 bootstrap replicates. To retain the well-conserved regions of the aligned G domain, columns with >50% gaps were removed, as identified using Consensus Finder [83]. The tree was inspected by eye to identify clades representing distinct subfamilies of trGTPases.

### Sensitive sequence searching and classification using HMMs

Multiple sequence alignments were used to make subfamily-specific Hidden Markov Models (HMMs) using HMMer 3.0 [124]. These served two purposes: firstly for sensitive sequence searching against a larger database of genomes, and secondly for classifying the resultant hits into subfamilies. Where curated alignments for specific subfamilies were available from my previous, published analyses (bacterial and organellar EF-G, Tet, Hbs1/eRF3-like, SelB and IF2 families [34,49,74,83,99]), these were used as input to HMMer in the first round of searching. Otherwise, the full-length sequences from the clades of subfamilies identified in the preliminary tree were extracted and aligned separately. In the case of organellar and GTPBP trGTPases, family-level phylogenies were first remade with more homologous amino acid positions to improve resolution of subfamily divisions.

The collection of HMMs was used to search a large collection of genomes (103 eukaryotes, 1274 bacteria and 105 archaea). The hits were inspected to identify the E value at which other GTPases are hit that are not part of the trGTPase superfamily based on the presence of the classical trGTPase G domain [8]. This value (e-20) was used as the cutoff for trGTPase superfamily membership. Thus although some more distantly related GTPases also appear to have roles in translation, for example ObG and HflX, these were not included in the current analysis.

The results of the HMM search were stored in a MySQL database and trees of the superfamily were remade as above to refine subfamily classifications. Predictions of localization to subcellular compartments was made with TargetP [125]. As all identified trGTPases were found to carry the G domain and domain II-homologous regions, the sequence region used to build the MAFFT alignment on which the superfamily tree was built was extended to encompass both these domains (an average length of 331 amino acids). This region begins at the N terminal boundary of the Pfam GTP_EFTU domain and ends at the C terminal boundary of the predicted D2 domain, or since not all trGTPases have a predicted Pfam D2 domain even though they do in fact carry it, 100 amino acids after the C terminal boundary of the GTP_EFTU (the average length of D2 is 103 amino acids). Alignments and HMMs of each identified subfamily were remade to update the HMM collection. The alignments were manually curated to remove fusion sequences (such as some Tet and CysN sequences) to avoid hits to non-trGTPase protein families. Duplicate sequences were also removed.

From this collection of HMMs, the genomes were rescanned, and the final classifications of 57 subfamilies were made. Classifications were made firstly by E value, and then corrected by taxonomy where E value could not reliably discriminate between groups (for example in the case of several chloroplast-encoded subgroups; see Results and discussion). In cases where an organellar subfamily HMM hits a bacterial trGTPases with greater significance than that of the general bacterial model for that subfamily, the predicted subfamily is recorded in Additional file 2 in the following format: “organellar subfamily (bacterial equivalent)”. For example, the EF-Tu from the cyanobacterium *Nostoc azollae* is classified by HMMs as cEFTu, so is recorded in Additional file 2 as “cEFTu(EF-Tu).”

The classification here does not always imply monophyly. Paraphyly is common in the superfamily, with one subfamily apparently arising from within another. This is especially the case with organellar sequences, that are often nested within sequences from bacteria, and duplicates of EF-G that have arisen in particular lineages of bacteria [83].

### The final superfamily tree

trGTPases from a representative selection of taxa were aligned with MAFFT using the L-INSI-I strategy and used to generate a superfamily tree. To avoid severe long branch attraction, the most divergent subfamilies (Ski7, aSelBL and all apicoplast-specific subfamilies) were not included in this tree. Otherwise, RAxML phylogenetic analysis of the G domain and Domain II-containing sequence region was carried out as above. The input alignment contained 239 aligned amino acid positions from 768 sequences.

## Additional files

Additional file 1:
**Table of final HMM search results.** The unique sequence identifiers are included for retrieval of data from online repositories. Additional file 2:
**Table of subfamilies mapped on to taxonomy.** Data includes final HMM search results, along with curated notes. Additional file 3:
**Table of synonyms for trGTPase subfamilies.** As there are inconsistencies in naming of trGTPases in the literature, this table shows the alternative names by which some trGTPase subfamilies are known. Additional file 4:
**Phylogeny of select EF1 family members.** Maximum likelihood phylogeny of relatively conservatively evolving (as indicated by branch lengths) subfamilies of the EF1 family. Numbers of branches show bootstrap support from 100 replicates. Branch lengths are proportional to the number of amino acid substitutions (see lower scale bar). Additional file 5:
**Phylogeny of GTPBP-type proteins from eukaryotes and archaea.** Maximum likelihood phylogeny of GTPBP from across the tree of life. Numbers of branches show bootstrap support from 100 replicates. Branch lengths are proportional to the number of amino acid substitutions (see lower scale bar). Additional file 6:
**Consensus alignment of the G domain and domain II.** A 60% consensus alignment of trGTPase subfamilies from Figure 1. Lines beneath the alignment show the locations of the P loop, Switch I-II and the GTP binding loop motifs (G1-5). The colored ruler shows the domains (red: GTPase, blue: domain II). The G’ and G” subdomains are indicated by pale green and yellow highlighting, respectively. Red triangular markers show sites involved in monovalent cation binding. Additional file 7:
**Table of subcellular targeting predictions.** The TargetP output is shown, with key below. Additional file 8:
**Text file of Tet proteins that contain the NYN_YacP RNase-like domain.** The sequences are in Fasta format. Additional file 9:
**Table of query sequences for the initial BlastP searches.** Links to sequences in web repositories and literature references are included. Additional file 10:
**Table of target organisms for the initial BlastP searches.** Taxonomic information is also recorded.
