# Supplementary figures and images for: The evolutionary and functional diversity of classical and lesser-known cytoplasmic and organellar translational GTPases across the tree of life
Source: BMC Genomics. 2015 Feb 14;16(1):78. doi: 10.1186/s12864-015-1289-7 (PMC4342817; doi:10.1186/s12864-015-1289-7)

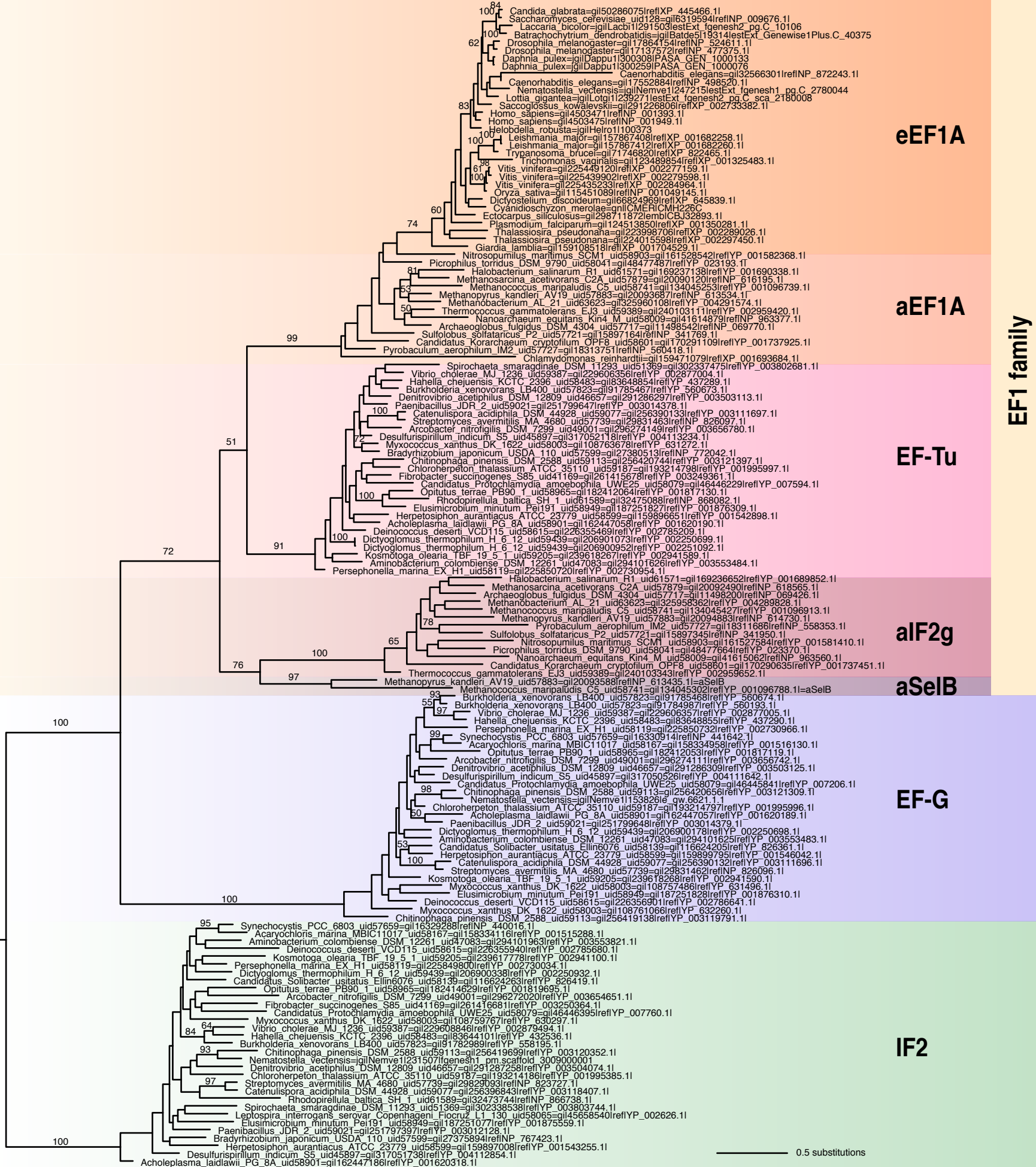

eEF1A

aEF1A

EF-Tu

aIF2g

aSelB

EF-G

IF2

EF1 family

0.5 substitutions

Supplement: Additional file 4: — Phylogeny of select EF1 family members. Maximum likelihood phylogeny of relatively conservatively evolving (as indicated by branch lengths) subfamilies of the EF1 family. Numbers of branches show bootstrap support from 100 replicates. Branch lengths are proportional to the number of amino acid substitutions (see lower scale bar). [file 12864_2015_1289_MOESM4_ESM.pdf]

eGTPBP1

eGTPBP2

eGTPBP

aGTPBP

Ascomycota eGTPBP2

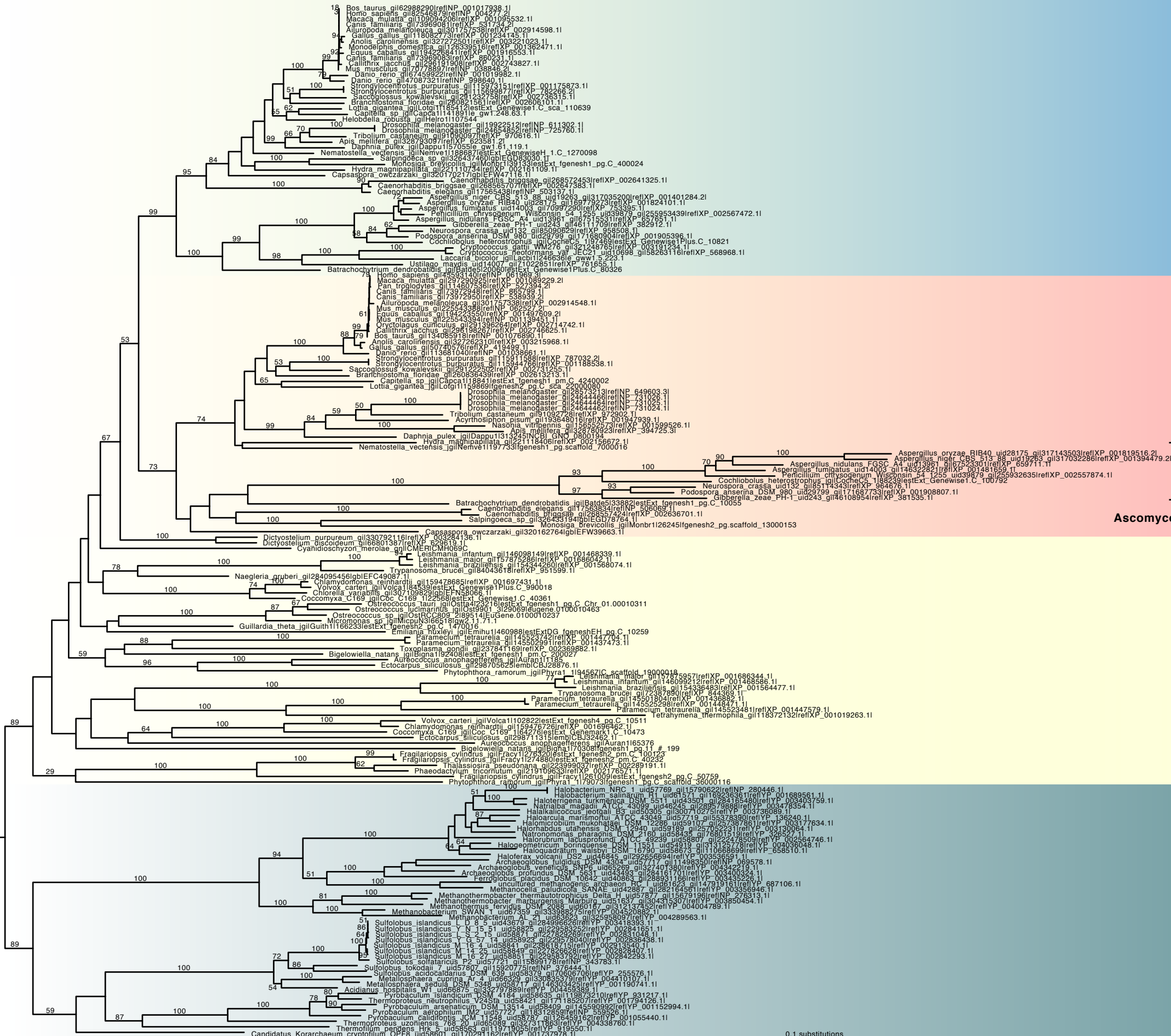

0.1 substitutions

Supplement: Additional file 5: — Phylogeny of GTPBP-type proteins from eukaryotes and archaea. Maximum likelihood phylogeny of GTPBP from across the tree of life. Numbers of branches show bootstrap support from 100 replicates. Branch lengths are proportional to the number of amino acid substitutions (see lower scale bar). [file 12864_2015_1289_MOESM5_ESM.pdf]
