# Supplementary material for: The evolutionary and functional diversity of classical and lesser-known cytoplasmic and organellar translational GTPases across the tree of life
Source: BMC Genomics. 2015 Feb 14;16(1):78. doi: 10.1186/s12864-015-1289-7 (PMC4342817; doi:10.1186/s12864-015-1289-7)
Supplement: Additional file 6: — Consensus alignment of the G domain and domain II. A 60% consensus alignment of trGTPase subfamilies from Figure 1. Lines beneath the alignment show the locations of the P loop, Switch I-II and the GTP binding loop motifs (G1-5). The colored ruler shows the domains (red: GTPase, blue: domain II). The G’ and G” subdomains are indicated by pale green and yellow highlighting, respectively. Red triangular markers show sites involved in monovalent cation binding. [file 12864_2015_1289_MOESM6_ESM.pdf]

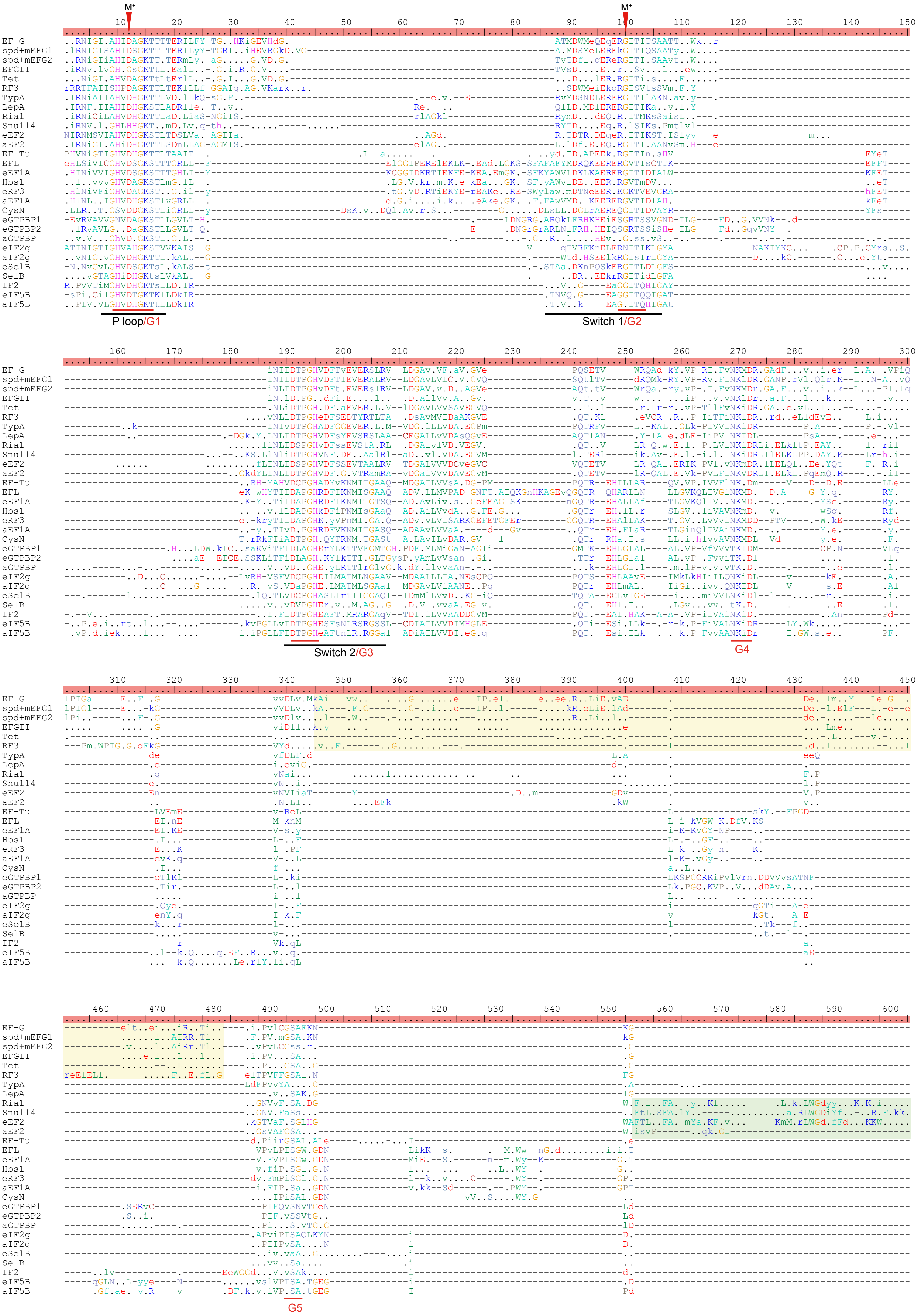
M+
M+
P loop/G1
Switch 1/G2
Switch 2/G3
G4
G5
EF-G
spd+mEFG1
spd+mEFG2
EFGII
Tet
RF3
TypA
LepA
Ria1
Snu114
eEF2
aEF2
EF-Tu
EFL
eEF1A
Hbs1
eRF3
aEF1A
CysN
eGTPBP1
eGTPBP2
aGTPBP
eIF2g
aIF2g
eSelB
SelB
IF2
eIF5B
aIF5B

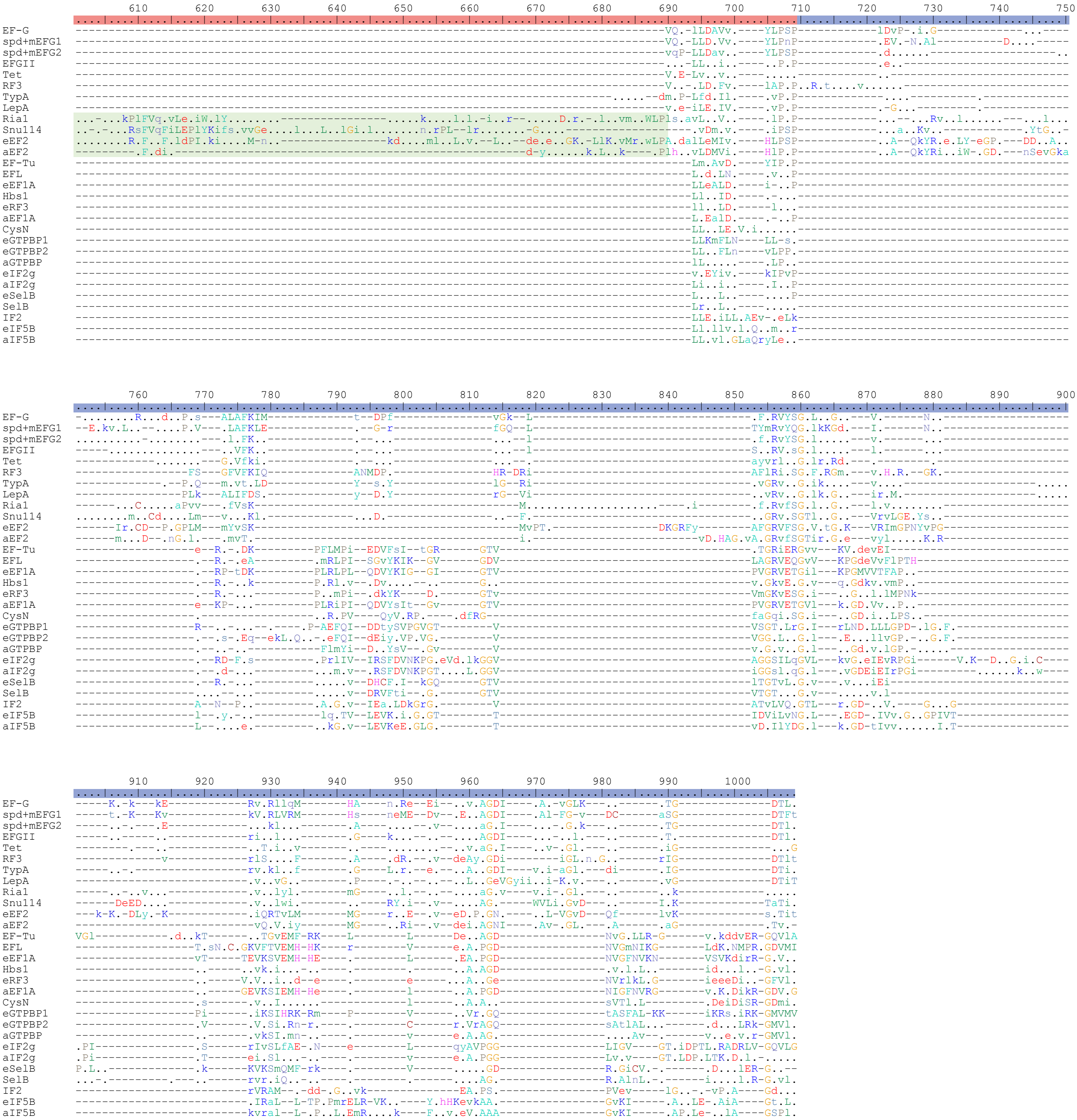

610 620 630 640 650 660 670 680 690 700 710 720 730 740 750
EF-G
spd+mEFG1
spd+mEFG2
EFGII
Tet
RF3
TypA
LepA
Ria1
Snu114
eEF2
aEF2
EF-Tu
EFL
eEF1A
Hbs1
eRF3
aEF1A
CysN
eGTPBP1
eGTPBP2
aGTPBP
eIF2g
aIF2g
eSelB
SelB
IF2
eIF5B
aIF5B
760 770 780 790 800 810 820 830 840 850 860 870 880 890 900
910 920 930 940 950 960 970 980 990 1000
